# The Treatment-induced Neuropathy Assessment Scale (TNAS): a psychometric update following qualitative enrichment

**DOI:** 10.1186/s41687-020-0180-8

**Published:** 2020-02-19

**Authors:** Tito R. Mendoza, Loretta A. Williams, Qiuling Shi, Xin Shelley Wang, Oluwatosin Bamidele, Jeanie F. Woodruff, Charles S. Cleeland

**Affiliations:** 1 Department of Symptom Research, The University of Texas MD Anderson Cancer Center, 1515 Holcombe Boulevard, Unit 1450, Houston, TX 77030 USA; 2 The University of Texas School of Public Health, Houston, TX USA; 3 Texas Heart Institute, Houston, TX USA

**Keywords:** Peripheral neuropathy, Patient-reported outcomes, Validation, Multiple myeloma, Colorectal cancer, Gynecological cancer, Chemotherapy-induced peripheral neuropathy, CIPN

## Abstract

**Background:**

The validation of the Treatment-induced Neuropathy Assessment Scale (TNAS v2.0), a patient-reported outcome measure of symptoms associated with cancer treatment-induced peripheral neuropathy (TIPN), was previously reported. Further patient input (qualitative interviewing, cognitive debriefing) suggested that the measure should be modified to better reflect the TIPN experience. We report the performance of a revised version (TNAS v3.0) for assessing TIPN across cancer treatments. This TNAS version incorporates extensive patient input, in accordance with FDA guidance on the development of patient-reported outcomes measures. Patients with multiple myeloma, colorectal cancer, or gynecological cancer treated with bortezomib, oxaliplatin, or taxane–platinum combination therapy, respectively, completed the TNAS v3.0, European Organization for Research and Treatment of Cancer Chemotherapy-Induced Peripheral Neuropathy (EORTC-CIPN20), and a cognitive debriefing survey during a scheduled clinic visit. Patients also participated in in-depth qualitative interviews about their TIPN symptoms. The psychometric properties of the TNAS v3.0 were evaluated.

**Results:**

Cognitive debriefing survey results were summarized and showed that most patients found the items easy to complete, comprehensible, acceptable, and not redundant. A notable change from TNAS v2.0 was the separation of “numbness” from “tingling,” although these 2 items remained the most severe, followed by a new “pain” item. The Cronbach coefficient alphas for the 9-item TNAS were 0.88 and 0.90 at the first and second administrations, respectively, indicating good reliability. The test–retest reliability of the TNAS was 0.97. The correlation coefficients for the 9-item TNAS and the EORTC-CIPN20 were 0.69 for the sensory subscale, 0.70 for the motor subscale, and 0.32 for the autonomic subscale, indicating good validity.

**Conclusion:**

This psychometric evaluation showed that the TNAS v3.0 is valid and reliable. Further research is needed to determine clinically meaningful differences in TNAS v3.0 scores and demonstrate its responsiveness over time.

## Background

The symptoms associated with treatment-induced peripheral neuropathy (TIPN, often referred to as chemotherapy-induced peripheral neuropathy, or CIPN), such as pain, numbness, tingling, sensitivity to hot and cold temperatures, and diminished motor skills, are the unintended consequences of various cancer therapies, including newer chemotherapies and targeted therapies [1, 2]. As a result of these symptoms, many patients report impaired daily functioning and poor quality of life [3–6]. Because TIPN is often persistent, a way to systematically assess TIPN is needed in order to understand the increasing burden brought about by residual treatment-related neuropathic effects. A recent review suggests that, given the significant overlap in symptoms among various neuropathy etiologies, a single instrument by which to measure multiple neuropathies is reasonable, provided it has content validity [7].

To this end, a patient-reported outcome (PRO) measure, the Treatment-induced Neuropathy Assessment Scale (TNAS) was developed and validated [8]. The symptom items for the initial versions of the TNAS (v1.0 and v2.0) were generated by expert panels and literature reviews, as well as by patient debriefing of the initial item set [8]. Data analysis results from the initial validation study indicated that the TNAS demonstrated responsiveness, reliability, and validity and that specific sensory and motor deficits were often more bothersome to patients than pain.

The development and validation of PRO measures should be an iterative process [9] and, as new, pertinent information is generated, such measures may need to be revised. To ensure that the TNAS closely adheres to the US Food and Drug Administration (FDA)‘s 2009 guidance on establishing content validity for PRO measures used in labeling claims [10], we collected extensive input from patients to appropriately refine the instrument. On the basis of results from one-on-one qualitative interviews with patients receiving treatments known to induce TIPN, we modified TNAS v2.0 to reflect extensive patient input (TNAS v3.0) [11]. To ensure that patients understood the TNAS v3.0 items, we conducted cognitive debriefing of the TNAS v3.0 with a subset of patients. We then performed a psychometric validation study of the TNAS v3.0 in patients with multiple myeloma, colorectal, or gynecological cancer receiving bortezomib, oxaliplatin, or taxane–platinum cancer therapies, respectively. Here, we report the results from the cognitive debriefing survey, cognitive debriefing interviews, and psychometric analysis of the TNAS v3.0.

## Methods

### History and development of the TNAS

The 11-item TNAS v1.0 was developed from a list of candidate symptoms suggested by clinicians who saw patients being treated with neurotoxic agents and from literature review of existing PRO neuropathy scales [8]. The TNAS asks patients to rate the severity of their neuropathy-related symptoms in the last 24 h. The TNAS is scored on a 0–10 scale, with 0 = the symptom is not present and 10 = the symptom is as bad as you can imagine. On average, the TNAS takes < 2 min to complete. On the basis of cognitive debriefing results performed on the 11-item TNAS v1.0, 2 other items were added to form a revised version (TNAS v2.0) [8].

The TNAS v3.0 described herein is a revision of TNAS v2.0 that is based on results from one-on-one qualitative interviews with patients receiving treatments known to induce TIPN [11]. The content domain and item generation for the TNAS v3.0 relied heavily on the results of these qualitative interviews, during which patients reported neuropathy-related sensations and interference with daily activities in response to open-ended prompts. The detailed results of the interviews are reported elsewhere [11]. This method allows solicitation of patient input about which sensations and impaired functions matter most to patients, and it is consistent with the 2009 FDA guidance in terms of establishing the content validity of a PRO assessment tool [10].

Because pain is an important symptom for patients with TIPN [8] and because it was endorsed by 67% of patients in the qualitative interviews, we included a “pain” item in the TNAS v3.0. We also included the item “disturbed sleep,” which patients deemed to be important in the qualitative interviews. The TNAS v2.0 items “Swelling in hands/feet at its WORST,” “Sensations of electric shock at their WORST,” “Discomfort when touching things at its WORST,” “Discomfort when skin comes into contact with something (e.g., blanket, clothing) at its WORST,” and “Pain when touching cold things at its WORST” were rated lowest in terms of severity in the previous validation study [8], consistent with the qualitative interview results showing that these items were reported as sensations associated with neuropathy by fewer than 10% of patients. These items were therefore dropped. The remaining TNAS v2.0 items were retained, although some were modified slightly in response to the qualitative interview results. Thus, the TNAS v3.0 to be tested in this study had 10 items. Table 1 provides a description of the TNAS versions and the corresponding changes.

**Table 1:** The development of the TNAS and the accompanying changes

TNAS version	Number of items	Methods	Changes made	Next steps
1	11	Expert clinician input, literature review	Original TNAS	Cognitive debriefing and psychometric analysis
2^a^	13	Cognitive debriefing, psychometric analysis	Two items were added: • “Feeling of coldness in hands/feet/fingers at its WORST” • “Swelling in hands and feet at its WORST”	In-depth qualitative interviewing, literature review
3^b^	10	In-depth qualitative interviewing, literature review	Five items were removed: • “Sensations of pin/needles in arms/legs at their WORST” • “Sensations of electric shock at their WORST” • “Cramps in your hands/feet at their WORST” • “Discomfort when touching things at its WORST” •“Discomfort when your skin comes in contact with something at its WORST” One item was split into two: • “Numbness or tingling in your hands or feet at its WORST” became ◦ “Numbness in your arms, legs, hands or feet at its WORST” ◦ “Tingling in your arms, legs, hands or feet at its WORST” Two items were added: • “Trouble finding or wearing shoes at its WORST” • “Disturbed sleep at its WORST” Six items were modified: • “Pain when touching cold things at its WORST” became “Pain in your arms, legs, hands, or feet at its WORST” • “Hot or burning sensation in hands or feet at their WORST” became “Hot or burning sensations in your arms, legs, hands, or feet at their WORST” • “Feelings of coldness in hands, feet, or fingers at its WORST” became “Feelings of coldness in your hands or feet at its WORST” • “Trouble grasping small objects at its WORST” became “Difficulty using your hands or fingers at its WORST” • “Trouble walking due to loss of feeling in your legs or feet at its WORST” became “Trouble walking at its WORST” • “Difficulty with your balance due to loss of feeling in your legs or feet at its WORST” became “Trouble with your balance or falling at its WORST”	Cognitive debriefing and psychometric analysis
3	9	Cognitive debriefing and psychometric analysis	One item was removed: •“Trouble finding or wearing shoes at its WORST”	Cognitive debriefing and psychometric analysis

*Abbreviation*: *TNAS* Treatment-induced Neuropathy Assessment Scale

^a^Further details are described in Mendoza et al., 2015 [8]

^b^Further details are described in Williams et al., 2018 [11]

### Scoring the TNAS

The TNAS was designed to measure overall neuropathy caused by cancer treatment. Hence, a global score that is the arithmetic average of all the items can be computed, with a higher score indicating greater neuropathy. Two subscale scores can also be calculated to evaluate symptoms related to sensory or interference dimensions. The sensory subscale score was the mean of 6 sensory items: numbness, tingling, pain, hot or burning, feelings of coldness and disturbed sleep. The interference subscale score was the mean of 3 interference items: trouble walking, trouble with balance and difficulty using hands. We also created a composite score based on the average of the 3 most severe and prevalent symptoms. When calculating any global score by taking the arithmetic mean, > 50% of the items must have valid responses; otherwise, the global score is considered missing. Note that by taking the arithmetic average of all items in a set, we implicitly assume equal weights for each of the items. Although some items seem to contribute more than others, it has been shown that simple linear scoring is robust [12] and adequate for most purposes [13].

### Data collection and participants

For this cross-sectional study, data were collected from 60 patients with multiple myeloma, colorectal cancer, or gynecological cancer who were treated with bortezomib, oxaliplatin, or a taxane–platinum-based cancer therapy, respectively. The patients were approached at any point along the continuum of their treatment trajectory and were recruited if they answered “yes” to the question, “Are you experiencing any unusual feelings in your hands or feet related to therapy for your cancer?”. Patients gave written informed consent to participate in this study, which was approved by our Institutional Review Board. All participants were administered the 10-item TNAS v3.0 [11], the European Organisation of Research and Treatment of Cancer 20-item chemotherapy-induced peripheral neuropathy scale (EORTC-CIPN20) [14, 15], and a cognitive debriefing survey during their scheduled clinic visit. Patients also participated in a detailed cognitive debriefing interview after completing the TNAS v3.0.

For test–retest purposes, a paper copy of the TNAS v3.0 was given to the patient with instructions to complete it the following day and to mail it back to the study team using a prepaid-postage envelope. Because the neuropathic side effects from chemotherapy may increase over the treatment period, we chose to examine the stability of the instrument by specifying the second administration for the following day. Research coordinators called all patients via telephone as a reminder to fill out the TNAS the following day. To minimize variation due to mode of administration, patients used the same paper version that was administered during their clinic visit.

The EORTC-CIPN20 assesses chemotherapy-induced neuropathy-associated symptoms and functional limitations. The items are rated on a 0–4 scale and are divided into sensory, motor, and autonomic subscales. This questionnaire was selected because it is used frequently in the cancer population [16, 17].

Sociodemographic information (including birth date, sex, ethnicity, level of education, place of residence, marital status, and employment status) and clinical information (including primary diagnosis, concomitant disease, and treatment data) were collected from the patients’ medical records by research staff.

### Cognitive debriefing

Cognitive debriefing provides evidence to support the content validity of a measure [18, 19]. In this study, cognitive debriefing results were used to support decisions for modification, retention, or deletion of TNAS v3.0 items prior to psychometric analysis.

#### Cognitive debriefing survey

Patients completed the cognitive debriefing survey to give feedback about the relevance of the TNAS v3.0 items to the patient’s disease and treatment conditions and for item comprehension and clarity. The survey asked participants about the ease of completion, comprehensibility, acceptability, and redundancy of the TNAS v3.0 items and about the ease of use, ease of understanding, and their level of comfort in using the 0–10 numeric rating scale. Patients were asked for suggestions as to whether any items needed to be clarified to make them easier to understand. Finally, participants were asked to recommend additional items that were not asked but that they thought should be included, if any.

#### Cognitive debriefing interviews

We also conducted detailed cognitive debriefing interviews with all patients. The interviews were digitally taped and transcribed verbatim for analysis. Patients were asked what each question meant to them. They were prompted to describe what they were thinking of when they responded to the question. They were asked if the descriptor “arms, legs, hands, or feet” in the first 4 sensory questions was helpful. They were asked if the descriptor “hands or feet” in the question about feelings of coldness was sufficient. They were asked if “balance” and “falling” should be included in the same question. They were asked if they understood that “disturbed sleep” was related to the neuropathy only.

### Statistical analysis

We prespecified that we needed 20 patients from each cancer type to ensure adequate representation of patients with neuropathic symptoms. For the validation portion of the study, we were interested in detecting significant correlations between the TNAS score and the sensory and motor subscales of the EORTC-CIPN20 as a measure of concurrent validity. With a sample size of 60, this study has 81% power to detect a significant result, assuming that the hypothesized population correlation is 0.38 using a 2-tailed test with a significance level of 0.025 (to adjust for the 2 EORTC-CIPN20 subscale scores).

Data collected were used to evaluate content validity and item clustering, calculate internal consistency, demonstrate concurrent validity, and describe the prevalence and severity of neuropathic symptoms. In addition, comparison of data from the second administration of the TNAS v3.0 with data from the first administration was used to demonstrate the instrument’s test–retest reliability. Analyses were conducted using Statistical Package of the Social Sciences (SPSS) v22. Means, standard deviations (SDs), and percentages of missing data were computed for all TNAS v3.0 items. Analysis of variance was used to determine whether the treatment groups differ on their TNAS V3.0 and EORTC-CIPN20 subscale scores.

#### Cognitive debriefing analysis

Frequency analysis of objective questions and content analysis of the open-ended questions were performed on the cognitive debriefing survey responses. The number of patients understanding each item correctly was tallied. Transcripts of the cognitive debriefing interviews were analyzed descriptively using MAXQDA 12 software (MAXQDA, Berlin, Germany).

#### Psychometric analysis

Reliability refers to the extent to which a measure is able to yield consistent, reproducible results. Cronbach coefficient alphas were computed to estimate the internal consistency reliability of all TNAS items. The criterion for good internal consistency (reliability) is a Cronbach alpha ≥0.70. We calculated the intraclass correlations between all TNAS v3.0 items from the patient’s initial visit (first administration) and their mailed-in (second administration) questionnaires.

Criterion validity refers to the extent to which an instrument correlates with another instrument that measures a similar concept. To examine criterion validity, we correlated TNAS v3.0 scores with the subscale scores from the EORTC-CIPN20. In addition, the composite score based on the average of the 3 most severe and prevalent symptoms was correlated with EORTC-CIPN20 subscale scores and with the global TNAS score.

## Results

### Patient characteristics

Of 133 patients approached, 60 gave written informed consent to participate in the study. Reasons for exclusion of the remaining 73 patients included no neuropathy (*N* = 11), neuropathy not related to treatment (*N* = 19), patient refusal (*N* = 15), patient missed appointment (*N* = 12), study staff were unavailable (*N* = 13) and other reasons (*N* = 3). Of the 60 enrolled patients, 20 had multiple myeloma treated with bortezomib, 20 had colorectal cancer treated with oxaliplatin, and 20 had gynecological cancer treated with taxane–platinum therapy. Demographic and clinical characteristics of the sample are summarized in Table 2. Mean age ranged from 58 to 68 years by cancer group. There were more women than men, and the sample was predominantly non-Hispanic white. Most patients had good Eastern Cooperative Oncology Group performance status (0–1).

**Table 2:** Patient demographic and disease characteristics, by cohort

	All *N* = 60	Bortezomib *n* = 20	Oxaliplatin *n* = 20	Taxane–Platinum *n* = 20
	n (%)	n (%)	n (%)	n (%)
Women	46 (77%)	13 (65%)	13 (65%)	20 (100%)
White non-Hispanic	46 (77%)	14 (70%)	18 (90%)	14 (70%)
ECOG performance status < 2	57 (95%)	18 (90%)	20 (100%)	19 (95%)
	Mean (SD)	Mean (SD)	Mean (SD)	Mean (SD)
Age, years	62.82 (10.53)	68.0 (8.25)	58.3 (7.68)	62.2 (12.94)

*Abbreviations*: *ECOG* Eastern Cooperative Oncology Group, *SD* standard deviation

### Cognitive debriefing and item verification

All 60 participants completed the cognitive debriefing survey. We found that for most patients, TNAS v3.0 was generally comprehensible and well received. In particular, more than 90% of the participants had no difficulty completing the questions or using the 0–10 rating scale. Furthermore, 82% of the participants did not find questions difficult to understand, and 97% did not find them repetitive.

Of the 14 patients who did not correctly understand the item “trouble finding or wearing shoes,” (Table 3), 3 expressed difficulty understanding the item and 11 described what the question meant, but that meaning did not match the intent of the TNAS developers, which suggested that inclusion of this item might not be appropriate [20]. Patients reported somewhat different understandings of the items “numbness” and “tingling.” Thus, we kept these items separated.

**Table 3:** Numbers and percentages of patients correctly understanding TNAS v3.0 questions in in-depth qualitative interviews (*N* = 30)

	Number	Percentage
1. **Numbness** in your arms, legs, hands, or feet at its WORST?	30	100
2. **Tingling** in your arms, legs, hands, or feet at its WORST?	29	97
3. **Pain** in your arms, legs, hands, or feet at its WORST?	28	93
4. **Hot or burning sensations** in your arms, legs, hands, or feet at their WORST?	26	87
5. Feelings of **coldness** in your hands or feet at its WORST?	27	90
6. **Difficulty using** your hands or fingers at its WORST?	26	87
7. Trouble **walking** at its WORST?	28	93
8. Trouble with your **balance or falling** at its WORST?	26	87
9. Trouble **finding or wearing shoes** at its WORST?	16	53
10. **Disturbed sleep** at its WORST?	29	97

*TNAS* Treatment-induced Neuropathy Assessment Scale

Detailed qualitative interviews were conducted for all 60 patients; however, we were satisfied that saturation had been reached after we had analyzed 30 interviews from consecutive patients within each treatment group (10 who had received bortezomib, 10 who had received oxaliplatin, and 10 who had received a taxane–platinum combination). With the exception of the item “trouble finding or wearing shoes,” the items were correctly understood by most of the patients (87%–100%) (see Table 3). Patients were able to describe situations that were appropriate to the intended meaning of the question if they had experienced that sensation or functional impairment related to neuropathy. Most patients (93%) felt that including the descriptor “arms, hands, legs, or feet” was helpful to let them know the areas of the body about which we were asking, although 10% did think it would be helpful to have separate questions for upper and lower extremities. Patients felt that mentioning specific areas of the body helped to focus their attention to better respond to the question. Most (77%) felt that only asking about “hands and feet” for feelings of coldness was appropriate, although a few (23%) did suggest adding “fingers and toes.” Only 2 patients (7%) suggested adding “feet” to the question about “difficulty using your hands or fingers,” because most thought that the “trouble walking” item covered trouble using the feet. Most patients (80%) agreed or were neutral about having a combined item for “balance or falling,” given that balance and falling are very closely related. Most patients (73%) understood that the “disturbed sleep” item referred to neuropathy interfering with sleep.

### Symptom severity

The item “trouble finding or wearing shoes” showed significant bias by sex, with a mean rating of 0.64 (SD 1.6) for men and 3.46 (SD 3.6) for women. On the basis of this result, along with results from the cognitive debriefing survey and in-depth interviews indicating that the item was not consistently understood (see Table 3), we dropped it from the TNAS v3.0.

Table 4 shows the summary statistics for the remaining 9 items of the TNAS v3.0. The number of missing TNAS items (3 overall) was minimal. Numbness and tingling were the 2 most severe symptoms at both assessment timepoints. In addition, these 2 items correlated with each other (0.83). Numbness and tingling were similarly correlated with overall TNAS score (0.75 vs 0.74). Numbness and tingling were also similarly correlated with the motor subscale of the EORTC-CIPN20 (0.56 vs 0.53). Numbness was slightly more correlated with the sensory subscale of the EORTC-CIPN20 than was tingling (0.71 vs 0.65).

**Table 4:** Summary statistics for the TNAS v3.0 items collected at the initial assessment, by cohort

	All *N* = 60			Bortezomib *n* = 20	Oxaliplatin *n* = 20	Taxane–Platinum *n* = 20
	Mean (SD)	Prevalence^a^	Percentage of Missing Items^b^	Mean (SD)	Mean (SD)	Mean (SD)
Numbness in arms, legs, hands, or feet	4.88 (2.8)	92%	0	3.65 (2.4)	5.00 (2.9)	6.00 (2.7)
Tingling in arms, legs, hands, or feet	4.69 (3.0)	92%	0.18%	3.70 (2.6)	4.60 (3.2)	5.84 (2.9)
Pain in arms, legs, hands, or feet	3.92 (3.5)	75%	0	3.25 (3.2)	2.95 (3.1)	5.55 (3.7)
Trouble walking	3.62 (3.2)	73%	0	2.60 (2.6)	3.05 (2.9)	5.20 (3.6)
Disturbed sleep	3.58 (3.3)	77%	0	3.55 (3.0)	2.70 (3.4)	4.50 (3.5)
Trouble with balance or falling	3.05 (3.1)	75%	0.18%	2.63 (2.5)	2.45 (2.9)	4.05 (3.6)
Feelings of coldness in hands or feet	2.95 (3.1)	67%	0	2.55 (2.8)	3.05 (3.1)	3.25 (3.4)
Hot or burning sensations in arms, legs, hands, or feet	2.59 (3.1)	58%	0.18%	2.11 (2.5)	1.75 (2.7)	3.90 (3.5)
Difficulty using hands or fingers	2.53 (2.9)	65%	0	2.10 (2.4)	2.35 (3.0)	3.15 (3.4)

*Abbreviation*: *TNAS* Treatment-induced Neuropathy Assessment Scale

^a^ Percentage of patients rating ≥ 1 on a 0–10 scale

^b^ On the 9-item TNAS, the denominator was 540 (9 item times 60 patients). Missing item responses were from 3 patients

Pain was the third-most-severe symptom in the initial assessment. Hot or burning sensations and difficulty using hands or fingers were rated as the least severe symptoms at both timepoints. All items had a prevalence of at least 58% (items with a severity of at least 1).

Table 5 shows that the treatment groups differ on their TNAS global score (*p* < .026) and sensory subscale scores (*p* < .025). However, no statistically significant difference was found on the EORTC-CIPN20 subscale scores.

**Table 5:** Summary statistics for the TNAS v3.0 and CIPN20 subscales at the initial assessment, by cohort

	Treatment Groups			
	Bortezomib *n* = 20	Oxaliplatin *n* = 20	Taxane–Platinum *n* = 20	*p*-value^*^
TNAS V3.0	Mean (SD)	Mean (SD)	Mean (SD)	
TNAS global	2.90 (1.51)	3.10 (2.09)	4.60 (2.56)	0.026
TNAS sensory	3.13 (1.63)	3.34 (2.04)	4.84 (2.55)	0.025
TNAS interference	2.40 (2.04)	2.62 (2.5)	4.13 (2.97)	0.070
EORTC-CIPN20^**^				
CIPN20 sensory	17.15 (4.74)	18.05 (5.48)	19.85 (7.03)	0.336
CIPN20 motor	13.45 (4.11)	12.95 (3.65)	14.35 (6.40)	0.656
CIPN20 autonomic	3.80 (1.4)	3.90 (1.37)	3.20 (1.40)	0.236

*Abbreviation*: *TNAS* Treatment-induced Neuropathy Assessment Scale, *EORTC-CIPN20* European Organization for Research in the Treatment of Cancer – Chemotherapy –Induced Peripheral Neuropathy

^*^ *P*-values based on one way analysis of variance comparing the three treatment groups

^**^EORTC-CIPN20 scores are based on raw scale scores

### Psychometric properties of the TNAS v3.0

The Cronbach coefficient alphas for the 9-item TNAS were 0.88 and 0.90 at the first and second administrations, respectively, indicating good reliability. The test–retest reliability of the TNAS calculated using interclass correlation was 0.97.

The correlation coefficients for the 9-item TNAS and the EORTC-CIPN20 were 0.69 for the sensory subscale, 0.70 for the motor subscale, and 0.32 for the autonomic subscale, indicating good validity. The correlation between the composite score based on the average of the 3 most-severe and prevalent symptoms (numbness, tingling, and pain) and the global TNAS score was 0.88. Correlations between the same composite score and the EORTC-CIPN20 were 0.71 for the sensory, 0.62 for the motor, and 0.22 for the autonomic subscales.

## Discussion

An existing measure of TIPN, the TNAS v2.0, was modified to incorporate extensive patient input via in-depth qualitative interviewing and cognitive debriefing. The items in this revised version, the TNAS v3.0, have both test–retest and internal consistency reliability and demonstrated concurrent validity.

A strength of this study is that 3 cancer groups (multiple myeloma, colorectal cancer, gynecological cancer) treated with different neurotoxic therapies (bortezomib, oxaliplatin, or a taxane–platinum combination) were included. This enabled the comparison of TNAS responses in patients with different primary diagnoses and exposure to different neurotoxic therapeutic agents. The FDA recommends a thorough literature review, patient input, and expert opinions as methods to establish content validity in the development of PRO measures. In a systematic review of 18 measures of chemotherapy-induced peripheral neuropathy, Gewandter et al. [7] found that the use of all 3 methods to establish content validity was reported for only 2 measures (NeuroQOL and EORTC-CIPN20).

Our present study reexamined the psychometric properties of the TNAS v3.0 after incorporating extensive input from patients with TIPN in the development and modifications of items. A notable change from the TNAS v2.0 was the separation of the items “numbness” and “tingling” on the basis of patients’ having somewhat different understandings of these 2 items. In addition, the individual items correlated with the similar items from the EORTC-CIPN20.

Patient input via cognitive debriefing showed that the revised and modified items were understandable, clear, and nonrepetitive. We removed the item “Trouble finding or wearing shoes at its WORST” because of large sex differences in the mean score and the difficulty reported by many of the patients in understanding the intended meaning of the item. Cognitive debriefing also demonstrated that the TNAS v3.0 was well understood and favorably received. Although most of the existing measures for neuropathy use verbal descriptor scale items [7], more than 90% of the participants in our study did not report any difficulty in using the 0–10 scoring system.

The 9 items comprising the TNAS V3.0 showed good internal consistency, with Cronbach coefficient alpha values of ≥0.78 indicating acceptable reliability. The intraclass correlation as measure of test–retest reliability was very reasonable, with a value > 0.90. For test–retest reliability, patients completed the TNAS using the same mode of administration (paper and pencil) but different locations (test in clinic and retest at home). This result suggests that location of administration does not have an effect on TNAS ratings. Correlations between the 9-item TNAS and the EORTC-CIPN20 were 0.69 for the sensory, 0.70 for the motor, and 0.32 for the autonomic subscales. Correlations of 0.69 and 0.70 represent overlap in dimensions. According to Cohen’s effect size convention, these represent medium to large effect sizes [21]. We are using the EORTC-CIPN20 as another measure of neuropathy. Both TNAS and CIPN20 are intended to measure the same construct of treatment-induced neuropathy. Based on our qualitative interviewing [11], the autonomic side effects were not prominent, and no items to measure these side effects were included in the TNAS. As expected, the correlation of 0.3 between the TNAS and the autonomic subscale of the CIPN20 was low.

We were also interested in how a composite score based on the average of the 3 most severe and prevalent TIPN items (numbness, tingling, and pain) would perform. The correlation between this composite score and the global TNAS v3.0 score was 0.88, suggesting that these 3 items explained 77% of the variability for all the items in the instrument. It was also reasonable and expected that this same composite score would correlate most with the sensory subscale, rather than the motor subscale, of the EORTC-CIPN20. This suggests that a composite score of these 3 items may be sufficient for clinical practice or for monitoring patients for the development of TIPN when neurotoxic therapies are administered. It is worth noting that the MD Anderson Symptom Inventory [22], a multisymptom measure used in clinical trials and practice, also asks patients to rate their numbness or tingling (as 1 item) and pain among a list of 13 symptoms. We plan future studies to examine how well these 2 items predict the overall TNAS score.

This study had limitations. First, our sample size was not large enough to perform multivariate analyses, particularly factor analysis; however, factor analysis of the TNAS v2.0 identified sensory and interference (motor functioning) dimensions as underlying factors [8]. Although we were unable to perform factor analysis with our current dataset, the previously identified factors were confirmed by patients in the qualitative interviews. Second, the design of the present study did not allow us to test the responsiveness of the TNAS v3.0. However, the TNAS v2.0 was shown to be sensitive to expected changes in clinical status [8]. The 9-item TNAS v3.0 will need to be tested longitudinally in patients at risk for neuropathy. The patients in the current study were receiving neurotoxic therapy; thus, it would also be helpful to administer the TNAS v3.0 to a group of patients whose treatment was reduced or discontinued because of neuropathy as a side effect of therapy.

## Conclusion

We modified a previously validated TNAS version, supported by extensive patient input. Patient input into the experience of TIPN is a strength of the resulting TNAS v3.0, because many existing measures of peripheral neuropathy were not established on the basis of systematic patient input or evaluation of their content validity. We also demonstrated the reliability and validity of the TNAS v3.0 in a new sample of patients receiving treatments known to be associated with peripheral neuropathy. Our analysis suggests that the TNAS v3.0 is an informative, useful PRO assessment tool that incurs little patient burden.

Validation is an iterative process. As data accumulate on a measure under study, we have a better understanding of how that measure performs. The TNAS v3.0 may be of practical use in multisite clinical trials because eliciting patient responses can be conducted remotely, particularly in studies where detailed neurological work-up for neuropathy is not available.

## Data Availability

Data on file, MD Anderson Cancer Center.

## Abbreviations

EORTC-CIPN20: European Organization for Research and Treatment of Cancer Chemotherapy-Induced Peripheral Neuropathy
FDA: US Food and Drug Administration
PRO: Patient-reported outcome
SD: Standard deviation
TIPN: Treatment-induced peripheral neuropathy
TNAS: Treatment-induced neuropathy assessment scale
